# IVF laboratory COVID-19 pandemic response plan: a roadmap

**DOI:** 10.1186/s43043-020-00043-2

**Published:** 2020-10-08

**Authors:** Fadi Choucair, Nagham Younis, Alia Hourani

**Affiliations:** 1 Middle East Fertility Society Embryology Specialty Interest Group, Beirut, Lebanon; 2 American University of Beirut Medical Center, Beirut, Lebanon; 3 University of Jordan, Amman, Jordan; 4 Quttainah Medical Center, Kuwait City, Kuwait

**Keywords:** COVID-19, Pandemic, Response plan, In vitro fertilization laboratory, Clinical embryology, Andrology, Emergency

## Abstract

**Background:**

The potential of COVID-19 severe pandemic necessitates the development of an organized and well-reasoned plan for the management of embryology/andrology laboratories while safeguarding the wellbeing of patients and IVF staff.

**Main body:**

A COVID-19 pandemic response plan was proposed for embryology and andrology laboratories for pre-pandemic preparedness and pandemic management in anticipation of a possible second coronavirus wave. Preparation involves many plans and logistics before a pandemic risk rises. Many operational changes can be considered during the pandemic. This plan includes logistical arrangements, reducing labor needs, conserving supplies, and protective measures for embryologists and gametes/embryos.

**Conclusion:**

The unpredictable emergence of the COVID-19 pandemic dictates the need for a preparedness plan for embryology/andrology laboratories, which includes an action-oriented plan to secure the safety of all stakeholders.

## Background

In view of the recent worldwide COVID-19 disease and predictions suggesting the inevitability of future pandemics, the need for a disaster plan for IVF laboratories has become a compulsory requirement. COVID-19 is caused by a virus from the coronaviridae family, named severe acute respiratory syndrome coronavirus 2 (SARS-CoV-2) [1]. Coronaviruses can be transmitted through the air in respiratory droplets (> 5-μm diameter) and aerosols (≤ 5-μm diameter). Transmission is also believed to occur through direct (contaminated hands) or indirect (contaminated surfaces and fomites) physical contacts, leading to cross-infections and outbreaks [2]. According to the most recent evidence from the World Health Organization (WHO) and the US Centers for Disease Control and Prevention (CDC), the predominant transmission route for coronaviruses is through droplets as the virus is conveyed by exhaled air (through normal breathing, coughing or sneezing) rather than aerosols [3, 4]. These heavy droplets can deposit on surfaces amplifying the propagation of the virus. Surprisingly, recent data showed that the prevalence of asymptomatic individuals with COVID-19 may be as high as 50–75% which challenges the triage of infected individuals [5].

It is estimated that more than 25% of transmissions occur in the workplace [6]. The occurrence of asymptomatic coronavirus-positive patients and healthcare workers in IVF units may contribute to an environment rich in respiratory viruses, leading to potential outbreaks in the working place and spillover into the community. In this regard, it was reported that coronaviruses have been implicated in nosocomial community outbreaks originating from hospitals [7, 8].

### Potential biological transmission

Several molecular characteristics of SARS-CoV-2 may justify concerns about the potential susceptibility of gametes (semen, testicular tissue) and embryos to viral infection [9, 10]. Although not universally supported [11–13], recent reports confirmed the presence of the virus in the semen of infected patients [14]. These findings justify calls for more vigilance in the work environment and for the need to put in place stringent measures to protect embryologists from potentially infected gametes.

The possibility of coronavirus survival in liquid nitrogen is currently not known, but it is suggested that viruses are likely to survive the freeze/thaw process [15]. During cryopreservation of gametes/embryos in liquid nitrogen, straws or vials may leak or shatter causing liquid nitrogen spill and potential cross-contamination of samples. Using an experimental model, Bielanski and colleagues confirmed the occurrence of viral cross-contamination of bovine embryos stored in liquid nitrogen by bovine airborne viruses [16]. In addition, the sterility of factory-derived liquid nitrogen is often not guaranteed, and the risk of contamination by human pathogens may theoretically exist. In theory, some airborne contaminants may contaminate liquid nitrogen during the process of gas compression [16]. When cryotanks are opened, the nitrogen vapor mixes with the atmospheric air creating ice sediments which falls into the vessel and accumulates at its bottom. During aggregation, these ice crystals may entrap various airborne microbes (bacteria, fungi…) leading to possible contamination of the cryotanks [17]. It follows that the risk of contamination and cross-contamination of cryopreserved human material in liquid nitrogen by nosocomial coronaviruses is of legitimate concern.

### Occupational person-to-person transmission

Apart from direct viral transmission, the presence of patients and healthcare workers in a tight and closed working space in the presence of a variety of pathologies causes the fomites and the air to be loaded with potential viral contaminants. Occupational transmission in clinical laboratories is exceptional events rarely reported. At present no data are available for coronavirus outbreaks in IVF laboratories. Nevertheless, it is evident that in clinical laboratory settings, infection prevention and control measures are often designed to act on most pathogen’s modes of transmission.

### The professional societies response

Professional societies, such as the American Society for Reproductive Medicine (ASRM) and the European Society for Human Reproduction and Embryology (ESHRE), have strongly urged to stop fertility treatments during the COVID-19 pandemic for the protection of human life (i.e., conceptus). The majority of these societies provided guidance for IVF laboratories for the gradual resumption of activities throughout the pandemic [18–21]. As COVID-19 keeps on spreading comprehensively, escalating case importation from overseas or residual infected seeds coupled with the resumption of economic activities, a second wave of COVID-19 seems plausible.

To meet these challenges, the authors, on behalf of the Middle East Fertility Society Embryology group, developed a response plan to outline the essential services and delineate the proper means to ensure the sustainability of IVF laboratory services in anticipation of a second COVID-19 pandemic wave. To that end, the authors aimed to provide a practical roadmap for a preparedness and management plan for the protection of human life (gametes/embryos and staff). It should be noted that some points raised might represent standard of practice in IVF or basic requirements for IVF laboratories accreditation. It is important to emphasize nonetheless that while these recommendations constitute appropriate guidelines for IVF laboratory management during a COVID-19 pandemic, they are not designed to dictate a rigid all-exclusive plan.

## Main text

### Heightened planning and logistic preparation: initial onset of a pandemic

It is widely accepted that proper pre-pandemic planning should occur at an early level, as outlined in Table 1 and discussed later. Several initial logistic preparations should be in order before the occurrence of the crisis. Although most of these preparatory steps comply with the basic emergency plan of the IVF laboratory, some important elements may require further highlighting. Basically, and as part of routine infection control practices, the IVF staff should receive adequate education and proper training on conventional infection control practices and standard operating procedures (SOP) [22].

**Table 1:** Early-level response plan: pre-pandemic planning recommendations for embryology and andrology laboratories

Recommendation	Basis of evidence
Educate staff on disease transmission and mitigation methods	Recommendation based on consensus guidelines (CDC) [22]
Perform hand hygiene compliance observation audits	Recommendation based on a systematic review [23]
Consider additional vaccination for preventing respiratory illness	Recommendation based on the WHO position paper [24]
Perform fit testing for N95 masks	Recommendation based on expert opinion
Stock essential PPE supply	Recommendation based on expert opinion
Prepare staff emergency contact lists	Recommendation based on a committee opinion paper (SART, ASRM) and guidelines (ESHRE for good practice) [25, 26]
Update and maintain records on a remote server	Recommendation based a committee opinion paper (SART, ASRM) [25]
Dedicate a spare storage cryotank and sufficient liquid nitrogen supply	Recommendation based on accreditation standards (College of American Pathologists) [27]
Clean regularly the cryotanks	Recommendation based on risk assessment research [16]
Store spare gas cylinders	Recommendation based on usual practice

Hand hygiene compliance observation audits should be periodically performed with special focus on avoiding touching noses, mouths, and eyes [23].

In an effort to reduce the burden of the pandemic on the healthcare system, embryologists are encouraged to obtain additional vaccination namely for preventing respiratory illness caused by influenza and pertussis [24]. Personnel should also participate in a fit testing for N95 masks (FFP2 the European equivalent) which provides protection against aerosol-generated particles [28]. This test ensures that masks are properly sealed to the face.

As part of the main laboratory emergency plan, the IVF personnel should know whom to contact during an emergency crisis [25, 26]. Along this perspective, the laboratory supervisor should prepare staff emergency and suppliers contact lists and make them accessible to all staff. More importantly, duplicate records must be maintained on a secure web server and updated regularly [25].

The supervisor must also make sure to stock essential PPE supply. Facilities must consider storing cleaned spare gas cylinders in a ventilated storage room to avoid supply chain failure during pandemic times [29].

It should be noted that an on-site spare storage cryotank and reserve of liquid nitrogen (LN2) supply are critical points which constitute requirements for laboratory accreditation (College of American Pathologists (CAP) accreditation 2019 checklist; RLM.03944) [27]. The laboratory should have a written procedure to monitor and maintain adequate liquid nitrogen levels and temperatures (CAP accreditation 2019; RLM.03940). The monitoring can be through visual measurement, weight scaling, or use of an alarm system [30]. Cryopreservation consents should state the possibility of system failures during storage in case of disasters [25]. The cryotanks require periodic decontamination using approved laboratory detergents with adequate rinsing with distilled water [16]. The decontamination of cryotanks is recommended owing to the potential accumulation of sediments and contamination of liquid nitrogen [16]. However, this practice is optional and is not recommended by the regulating reproductive societies (ESHRE, ASRM, CAP).

### Operational changes during the pandemic stages

Due to the nature of coronavirus transmission, the WHO advocated and enforced “social distancing” measures to maintain the 2-m “safe” distance between people in addition to the routine use of PPE [31, 32]. As previously mentioned, the high proportion of asymptomatic individuals may imply that an infected asymptomatic embryologist may infect his/her colleagues if adequate protective measures and gears are not properly used. In addition, staff should be educated to recognize early signs of viral illness, namely sore throat, fever, and cough.

As numerous plans for work have been proposed by professional societies and regulations bodies across the globe during the pandemic period, it seems reasonable for healthcare workers to adopt these measures through normal times in anticipation of new pandemic waves.

In extreme scenarios, the complete closure of IVF clinics and the total cessation of all procedures are recommended in the event of a new pandemic. However, in the “new normal” operational practices, many interventions may be changed to adapt to the pandemic risk (Table 2).

**Table 2:** Pandemic response plan: recommendations for embryology and andrology laboratories during the coronavirus pandemic

Recommendations	Basis of evidence
**Operational changes reducing labor needs and conserving supplies**	
Identify essential the right people to fulfill the essential roles	Recommendation based on expert opinion
Create rotation shifts	Recommendation based on committee opinion guidelines (ESHRE COVID-19 taskforce) [18]
Stock PPE and cryopreservation medium and culture media batches favoring products with longer expiration dates	Recommendation based on expert opinion
Reduce the number of operated incubators	Recommendation based on expert opinion
**Operational changes protecting embryologists and gametes/embryos**	
Perform triage of staff and patients, and test according to the national recommendations and/or availability of laboratory testing	Recommendation based on committee opinion guidelines (ESHRE, ASRM COVID-19 taskforce) [18, 19]
Use proper PPE (surgical masks, gloves) or N95 masks when performing an aerosol-generating procedure	Recommendation based on experimental research [28]
Wear a surgical mask and practice social distancing at all time	Recommendation based on public health epidemiologic data [32]
Postpone visits for cryopreservation storage renewal payment and allow online billing using an electronic medical record system	Recommendation based on expert opinion
Perform cryopreservation consenting online	Recommendation based on expert opinion
Cleaning practices must be performed by laboratory staff (surfaces, keyboards, doorknobs, supplies boxes…)	Recommendation based on usual practice
Continuing positive-pressure air purification, reducing air recirculation, and favoring 100% fresh air	Recommendation based on expert opinion
Spray and wipe with approved disinfectant followed by UV irradiation for 30 min the workspace in the biosafety cabinet	Recommendation based on experimental research [33]
Wash thoroughly cumulus-oocyte complexes to potentially dilute the viral load	Recommendation based on expert opinion
Use protective eyewear and N95 masks when performing aerosol-generating procedures	Recommendation based on expert opinion
Semen ejaculate jars must be wiped prior to handling specimens	Recommendation based on expert opinion
Process sperm using a combination of sperm density gradient techniques followed by a swim-up step	Recommendation based on expert opinion
Collect the follicular fluid into well-sealed containers	Recommendation based on committee opinion guidelines (ESHRE COVID-19 taskforce) [18]
Use mechanical micropipettes and filtered tips. Following use, disassemble and disinfect micropipettes using approved laboratory agents and/or UV irradiation	Recommendation based on usual practice
Use UV-sterilized liquid nitrogen	Recommendation based on risk assessment research [34]
Use of closed system cryopreservation tools	Recommendation based on committee opinion guidelines (ESHRE COVID-19 taskforce) [18]
Safeguard the integrity of the zona pellucida	Recommendation based on experimental research [35]

#### Operational changes reducing labor needs and conserving supplies

Several operational changes should be implemented in the IVF laboratory in order to maintain the continuity of care by ensuring the safety of the personnel and avoiding the shortage in supplies.

To meet these goals, laboratory managers are expected to identify the right people to fulfill the essential roles. In spite of a forced shut-down policy during a pandemic, some procedures such as oocyte vitrification for oncology patients are deemed to be of urgent nature. In such cases, the supervisor should also explore the option of hiring free-lancers to fulfill essential tasks. In other words, the number of embryologists reporting to work should be reduced to a minimum without compromising safety and quality. Embryologists would be expected to perform rotating shifts depending on the size of the unit and staff. The rotating schedule is created to minimize exposure time to infection while accommodating the continuity of care within the laboratory [18].

During a pandemic, manufacturing and distributing companies may lack the personnel to maintain a regular delivery schedule. This shortage would require the laboratory to increase preemptively its stock of PPE (gloves, surgical masks, and N95 masks) as well as culture and cryopreservation media batches while favoring products with longer expiration dates.

Along this perspective, supplies inventory should be updated regularly in order to match the workload. A reduction in the workload may also drive special considerations for resources management, such as the reduction of the number of operating incubators.

#### Operational changes protecting embryologists and gametes/embryos

It is reasonable to recommend that patients and staff be primarily triaged and regularly tested according to national recommendations and/or availability of laboratory testing. While ESHRE favored SARS-CoV-2 IgM/IgG immunologic screening, ASRM emphasized on viral molecular testing [18, 19]. One should never underestimate the importance of personal hygiene and the use of proper PPE. Laboratory staff are expected follow standard laboratory practices, including wearing surgical masks, disposable gloves, and eye protection devices. It would be reasonable to add the use of a long-sleeve impermeable gown over the scrubs when performing an aerosol-generating procedure such as testicular tissue processing. In vitro experimental studies investigating the filtering capacity of standard surgical masks and N95 masks (FFP2) [28] showed that both types of masks retained at least 95% of small particles (0.3 μm corresponding to aerosols) offering comparable effective outward protection. Toward inward protection nonetheless was superior for N95 masks. It follows that while surgical masks may not be a perfect match to N95 masks, their usage is still encouraged. While the use of surgical masks is currently standard practice, N95 masks may be encouraged during aerosol-generating procedures. In view of the global shortage in facial masks during the COVID-19 pandemic, recommendations have been modified in order to take into account the local availability of PPE supply

It also follows that unnecessary patient visits to the IVF unit for the renewal of cryopreserved biological material should be reduced. Alternatively, payments and consents may be performed online. IVF staff should wear a surgical mask and practice social distancing at all times, more so when communicating information to patients. The role for telehealth is critical during pandemic time, and has been facilitated by the widespread use of Electronic medical record (EMR) systems for tasks including online billing, e-consents, and communications [36].

When discussing indoor laboratory air environment, no clear recommendations exist for the air purification system in IVF. Although high-efficiency particulate air (HEPA) filters are 99.97% efficient at collecting the most-penetrating particles (~ 0.3 μm), they may not adequately protect against smaller entities like viruses. But, since aerosolized viruses are thought to exist as agglomerates, the increase in the particle size may consequently cause them to be efficiently captured by the filter [37]. On the other hand, activated carbon filters normally installed within HVAC filter cartridges to trap volatile organic compounds, were found to absorb viral particles as well [38]. While most IVF laboratories are equipped with positive-pressure air systems, it may be recommended to switch to negative pressure modules during airborne virus outbreaks to sink the virus. This may be technically and financially challenging to many laboratories. In view of the restricted personnel number and laboratory space within the IVF working place, the risk benefit of discontinuing positive-pressure air purification must be properly assessed. The treatment of a patient who tested positive for COVID-19 for instance warrants the turning off of positive-pressure ventilation to avoid dissemination of the virus outside the operating theater. Particular consideration should also be given to increasing the number of air changes per hour and reducing recirculation of air in favor of using 100% fresh air.

As for infection control prevention, embryologists are encouraged to follow standard practices in the IVF laboratory. Such practices include the cleaning of supply boxes and gas cylinders before entering the laboratory using approved disinfection agents [21]. In addition, the biosafety cabinet workspace should be routinely and thoroughly disinfected, with particular attention to the uncluttering of airflow outlets to avoid flow disruptive turbulence, which may increase the risk exposure of operators. Specifically, personnel should exercise laboratory housekeeping vigilance during high-risk pandemics. Cleaning practices must be diligently observed, such as disinfection of common surfaces (e.g., doorknobs, keyboards, surfaces…). Commercially available quaternary-ammonium-based formulations, labeled “embryo-safe”, were shown to be effective against coronaviruses [39]. More so, the combination of spraying and wiping with approved disinfectant, followed by UV irradiation for 30 min may completely inactivate the virus in biosafety cabinets [33].

Since coronaviruses were detected in the blood of infected patients [40] and in view of the presence of viral receptors on human spermatozoa, Sertoli cells, Leydig cells [9, 10], and oocytes [41], extra vigilance during handling biological material is highly justified. More specifically, standard precautions should be observed during routine handling of specimens, while special attention should be considered during aerosol-generating procedures such as processing of testicular tubules. The use of N95 masks, goggles or face shields may be more appropriate during sample preparatory steps that may generate aerosols or droplets and during mechanical processing of testicular tubules.

It should also be noted that universal precautions during oocyte pick up should be strictly taken, which include the collection of follicular fluid into well-sealed containers [18]. In addition, cumulus-oocyte complexes should be thoroughly and repeatedly washed with the aim of diluting viral load. While no recommendations are currently available for sperm processing during COVID-19 pandemics, precautions and procedures recommended for other viruses should be diligently followed. Semen ejaculate jars must be properly wiped prior to handling specimens. It is advisable to separate sperm using a combination of sperm density gradient techniques followed by a swim-up step to dilute the virus [42]. Sperm processing should include the frequent change of sterile pipettes and tubes prior to each washing step [43]. The use of mechanical micropipettes is recommended [26] and sterile-filtered tips pipettes are preferred to avoid aerosol generation and contamination of the micropipette. Following use, micropipettes must be disassembled and disinfected using approved laboratory agents and/or UV irradiation.

The safety of the cryopreservation process may be best observed when UV-sterilized liquid nitrogen is used [34] and when embryos and oocytes are sequentially washed. Special attention should be made to safeguarding the integrity of the zona pellucida which is considered an efficient barrier against viral contamination [35]. The use of closed system cryopreservation tools is preferred to secure the safety of the storage material [18]. Vapor tanks are also favored for cryostorage to minimize risks of viral transmission.

## Conclusion

Planning for a crisis is one of the most important undertakings in a laboratory. Appropriate planning protects both personnel and gametes/embryos and ensures continuity of care. The planning process involves the prompt and proper identification of risks and the strict implementation of operational changes to address loss of staffing, shortage of supply, and changes in laboratory practices.

## Data Availability

Not applicable

## Abbreviations

ASRM: American Society for Reproductive Medicine
CAP: College of American Pathologists
CDC: Centers for Disease Control and Prevention
COVID-19: Coronavirus disease 2019
EMR: Electronic medical record
ESHRE: European Society for Human Reproduction and Embryology
FFP2: Filtering facepiece
HEPA: High-efficiency particulate air
IgG: Immunoglobulin G
IgM: Immunoglobulin M
IVF: In vitro fertilization
LN2: Liquid nitrogen
PPE: Personal protective equipment
SARS-CoV-2: Severe acute respiratory syndrome coronavirus 2
SOP: Standard operating procedure
UV: Ultraviolet
WHO: World Health Organization
